# *CONSTITUTIVE PHOTOMORPHOGENIC 10* (*COP10*) Contributes to Floral Repression under Non-Inductive Short Days in *Arabidopsis*

**DOI:** 10.3390/ijms161125969

**Published:** 2015-11-05

**Authors:** Min-Young Kang, Hye-Young Kwon, Na-Yun Kim, Yasuhito Sakuraba, Nam-Chon Paek

**Affiliations:** Department of Plant Science, Plant Genomics and Breeding Institute, Research Institute of Agriculture and Life Sciences, Seoul National University, Seoul 08826, Korea; mykang@snu.ac.kr (M.-Y.K.); kkoma1023@hanmail.net (H.-Y.K.); yunieie@snu.ac.kr (N.-Y.K.)

**Keywords:** *Arabidopsis thaliana*, flowering, *GIGANTEA* (*GI*), *CONSTITUTIVE PHOTOMORPHOGENIC 10* (*COP10*), protein-protein interaction, MSI4/FVE

## Abstract

In *Arabidopsis*, *CONSTITUTIVE PHOTOMORPHOGENIC/DE-ETIOLATED/FUSCA* (*COP/DET/FUS*) genes act in repression of photomorphogenesis in darkness, and recent reports revealed that some of these genes, such as *COP1* and *DET1*, also have important roles in controlling flowering time and circadian rhythm. The COP/DET/FUS protein COP10 interacts with DET1 and DNA DAMAGE-BINDING PROTEIN 1 (DDB1) to form a CDD complex and represses photomorphogenesis in darkness. The *cop10-4* mutants flower normally in inductive long days (LD) but early in non-inductive short days (SD) compared with wild type (WT); however, the role of *COP10* remains unknown. Here, we investigate the role of *COP10* in SD-dependent floral repression. Reverse transcription-quantitative PCR revealed that in SD, expression of the LD-dependent floral inducers *GI*, *FKF1*, and *FT* significantly increased in *cop10-4* mutants, compared with WT. This suggests that COP10 mainly regulates *FT* expression in a *CO*-independent manner. We also show that COP10 interacts with GI *in vitro* and *in vivo*, suggesting that COP10 could also affect GI function at the posttranslational level. Moreover, *FLC* expression was repressed drastically in *cop10-4* mutants and COP10 interacts with MULTICOPY SUPPRESSOR OF IRA1 4 (MSI4)/FVE (MSI4/FVE), which epigenetically inhibits *FLC* expression. These data suggest that COP10 contributes to delaying flowering in the photoperiod and autonomous pathways by downregulating *FT* expression under SD.

## 1. Introduction

Most flowering plants have evolved to synchronize their growth and development with seasonal environmental changes, especially changes in the intensity and period of daylight and temperature. The precise control of flowering time strongly affects regional adaptation and several signaling pathways, including photoperiod, vernalization, gibberellin, ambient temperature, and autonomous pathways, regulate floral induction [1]. Work to date has identified many genes that control flowering time and the major floral inducers *GIGANTEA* (*GI*), *CONSTANS* (*CO*), and *FLOWERING LOCUS T* (*FT*) have been studied widely and intensively [2,3,4]. The florigen FT acts in multiple flowering pathways [2,5]. CO protein directly binds to the *FT* promoter and upregulates *FT* mRNA, but this role differs somewhat under LD and SD conditions. Under LD conditions, *CO* expression coincides with light at the end of the day, and expressed CO protein promotes *FT* expression to induce flowering. Under SD, by contrast, the peak of *CO* transcription occurs after dusk and CO protein is unstable in the dark; therefore, CO cannot induce *FT* expression [6,7,8,9].

GI and FLAVIN-BINDING, KELCH REPEAT, and F-BOX PROTEIN1 (FKF1) have essential functions in the timing of daily *CO* expression. GI and FKF1 form a complex to destabilize CYCLING DOF FACTOR1 (CDF1), a key *CO* repressor [10,11]. Under LD conditions, expression of *GI* and *FKF1* peaks in the afternoon and the GI-FKF1 complex is recruited to the *CO* chromatin, where it degrades CDF1 to activate *CO* expression. Conversely, under SD, the peaks of *GI* and *FKF1* expression overlap less than they do in LD, leading to minimal formation of the GI-FKF1 complex [10,12]. This indicates that GI acts as a floral inducer with FKF1 in the *CO-FT* photoperiod pathway under LD. However, Sawa *et al.* (2011) reported that under SD conditions, the overexpression of *GI* increased *FT* expression without increasing *CO* expression; GI directly regulates *FT* expression by binding to the *FT* promoter region near the binding sites of *FT* repressors such as SHORT VEGETATIVE PHASE (SVP), TEMPRANILLO1 (TEM1), and TEM2 [13,14,15]. Thus, that GI regulates *FT* expression through both *CO*-dependent and *CO*-independent pathways. In addition, genes involved in the autonomous and vernalization pathways also regulate *FT* expression. *FLOWERING LOCUS C* (*FLC*) acts at a central place in the autonomous and vernalization pathways and FLC directly regulates *FT* and *SOC1* expression by binding to their promoters [13,16,17]. *FLC* expression is mainly regulated by histone modification factors [18]. For example, MULTICOPY SUPPRESSOR OF IRA1 4/FVE (MSI4/FVE) represses *FLC* expression by histone modification of *FLC* locus with DDB1 [19,20].

*CONSTITUTIVE PHOTOMORPHOGENIC/DE-ETIOLATED/FUSCA* (*COP/DET/FUS*) genes have important roles in the repression of seedling photomorphogenesis in darkness [21]. We recently reported that DE-ETIOLATED1 (DET1), a COP/DET/FUS family protein, negatively regulates flowering, because *det1-1* weak mutants (note that the *det1* null mutant is lethal) flower early, especially much earlier in SD, compared with the wild type [22]. In *det1-1* mutants, *FT* expression is significantly up-regulated in SD, but the expression levels of *GI* and *FKF1* do not change. DET1 physically interacts with GI to inhibit its binding to the *FT* promoter [22]. Furthermore, the expression level of *FLC*, a repressor of *FT*, is significantly down-regulated in *det1-1* mutants, probably due to the lack of interaction between DET1 and MSI4/FVE. Collectively, these observations indicate that DET1 acts in both photoperiod (post-translational regulation of GI) and autonomous (MSI4/FVE-FLC) pathways to repress the expression of *FT*. Similar to DET1, the COP/DET/FUS protein COP1 also regulates SD flowering in *Arabidopsis*; *cop1* mutants flowered much earlier than WT only under SD conditions [23]. COP1 is an E3 ubiquitin ligase and forms a complex with SUPPRESSOR OF PHYA1 (SPA1) for its E3 function [24]. The COP1-SPA1 complex is required for the ubiquitination and degradation of CO and GI in the night [7,23]. These results indicate that some COP/DET/FUS proteins also have important roles in repressing flowering, as well as the repression of photomorphogenesis in darkness. 

DET1 forms a multi-protein complex with COP10 and DAMAGED DNA BINDING PROTEIN1 (DDB1). The COP/DET/FUS family protein COP10 is an E2-like protein that lacks E2 activity [25]. DDB1 is required for the interaction of the COP10-DET1-DDB1 complex (termed the CDD complex) with CULLIN4 (CUL4), and acts as E3 ubiquitin ligase (CUL4-CDD E3 ligase) [26]. In addition, the CDD complex maintains the circadian rhythm with LHY and CCA1 in the photoperiod pathway [27,28]. These previous reports indicate that, like DET1, COP10 also affects the regulation of flowering time. However, the molecular mechanism of COP10 in flowering remains unclear.

In this study, we show that COP10 delays flowering time in SD by modulating GI at both transcriptional and post-translational levels in the photoperiod pathway. In addition, COP10 indirectly up-regulates *FLC* expression by interacting with MSI4/FVE, which functions in histone modification of the *FLC* locus. Our results show that COP10 functions in both the photoperiod and autonomous pathways to repress *FT* expression in SD.

## 2. Results and Discussion

### 2.1. GIGANTEA (gi-1) Is Epistatic to cop10-4 in the Photoperiodic Pathway of Flowering

**Figure 1:**
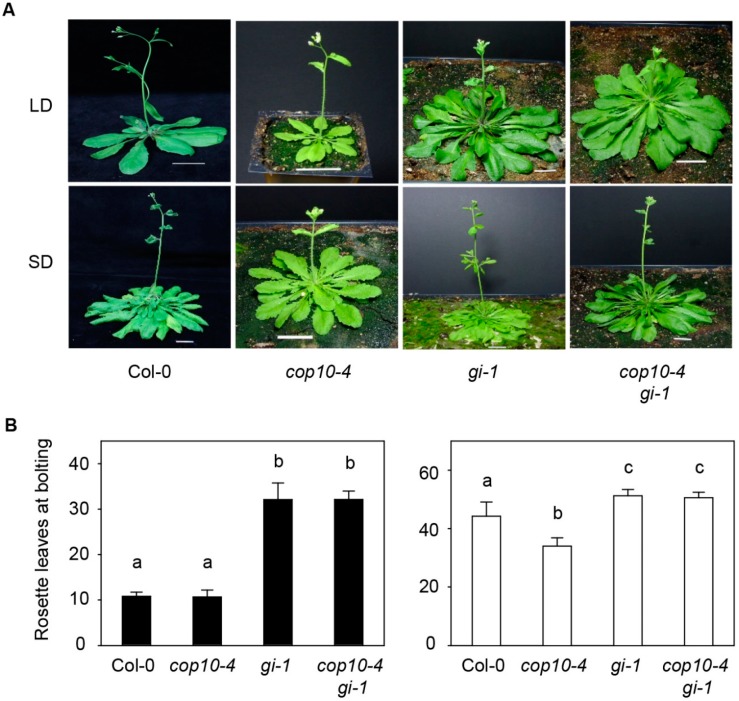
The early flowering phenotype of *cop10-4* and *gi-1* is epistatic to *cop10-4*. Phenotypes (**A**) and the number of rosette leaves (**B**) of wild type (WT, Col-0 ecotype), *cop10-4*, *gi-1*, and *cop10-4 gi-1* mutants in long days (LD) (black bars) and short days (SD) (white bars) Plants were grown at 22 °C under cool-white fluorescent light (90–100 μmol·m^−^^2^·s^−^^1^) in LD (16-h light:8-h dark) or SD (10-h light:14-h dark), and photographed at two to four days after bolting. Scale bars = 2 cm; and (**B**) Flowering time was measured as the number of rosette leaves at bolting. Means and standard deviations were obtained from more than 20 plants. These experiments were repeated three times with the same results. Bars with different letters are significantly different according to Duncan’s multiple range test (*p* < 0.05).

*cop10-4* has been isolated as one of the *cop10* mutant alleles, which has a mutation in the second exon of COP10, resulting in a weak allele although transcriptional and protein levels of COP10 do not alter in *cop10-4* mutant [25]. *cop10-4* mutants show photomorphogenic development in darkness, with phenotypes such as short hypocotyls and opened cotyledons [29,30,31,32]. However, the function of COP10 in the regulation of flowering time remains unexamined. Null mutants of *COP10* are lethal [25]; therefore, to examine the function of COP10 in flowering, we studied flowering time using the weak, viable allele *cop10-4*. We counted the number of rosette leaves (RLs) at bolting under both LD (16-h light:8-h dark) and SD (10-h light:14-h dark) conditions (Figure 1; Table S1). We found that under SD, *cop10-4* mutants flowered earlier, with 34.0 ± 2.8 RLs, about 10 fewer than wild type (WT, 44.3 ± 4.9 RLs). By contrast, WT and *cop10-4* flowered with almost the same number of RLs under LD, with 10.8 ± 0.9 RLs for WT and 10.7 ± 0.5 RLs for *cop10-4*, indicating that COP10 acts as a floral repressor mainly under non-inductive SD conditions, and plays an important role in photoperiod sensitivity in *Arabidopsis*.

DET1 acts as a floral repressor upstream of GI and attenuates binding of GI to the *FT* promoter [22]. COP10 interacts with DET1 and DDB1 to form a CDD complex, which represses photomorphogenesis in darkness [33]; COP10 could also act with GI in flowering. To test whether *COP10* interacts genetically with *GI*, we generated *cop10-4 gi-1* double mutants to compare with WT (and each single mutant) in flowering time under LD and SD conditions (Figure 1). As previously reported, *gi-1* mutants, which has 5-bp deletion resulting in premature stop codon, showed a strong late-flowering phenotype [3,31]; *gi-1* had 32.1 ± 3.7 RLs at bolting in LD and 51.2 ± 2.2 RLs in SD (Figure 1A,B). We found that *cop10-4* *gi-1* mutants showed a late-flowering phenotype, with 32.1 ± 1.8 RLs in LD and 50.6 ± 1.9 RLs in SD, almost the same as the *gi-1* mutant, but many more than the *cop10-4* mutant. These results indicate that *gi-1* is epistatic to *cop10-4* in the photoperiodic pathway of floral induction.

### 2.2. The cop10 Mutation Alters the Expression of Flowering Time Genes

**Figure 2:**
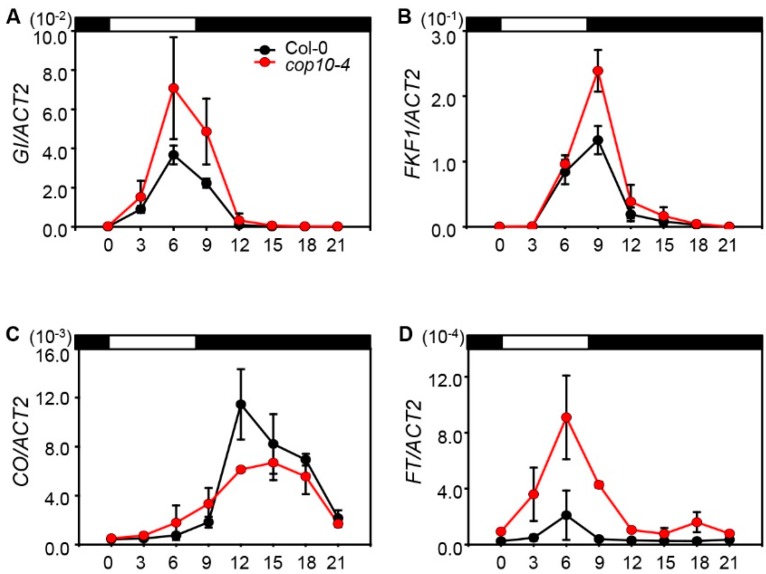
Effect of *cop10-4* on *GIGANTEA* (*GI*), F-BOX PROTEIN1 (*FKF1*), *CONSTANS* (*CO*), and *FLOWERING LOCUS T* (*FT*) expression under short days (SD). The expression of *GI* (**A**); *FKF1* (**B**); *CO* (**C**); and *FT* (**D**) was analyzed in Col-0 and *cop10-4* mutants by real-time PCR using three-week-old plants. Plants were grown at 22 °C under SD (8-h light:16-h dark) conditions, and plant tissues were harvested every 3 h. *ACT2* expression was used for normalization. Means and standard deviations were obtained from three biological replicates. These experiments were repeated twice with the same results.

To investigate the effect of *cop10-4* on the expression of floral inducers in the photoperiod pathway, we analyzed the phases and amplitudes of *GI*, *FKF1*, *CO*, and *FT* mRNA levels in WT and *cop10-4* mutants grown under SD conditions (Figure 2). In SD conditions, the transcript levels of *GI* and *FKF1* were the highest at ZT6 (zeitgeber time; 6 h after dawn) and ZT9, respectively [10]. We found that the transcript levels of *GI* at ZT6 and *FKF1* at ZT9 were almost the same in WT and *cop10-4* mutants. However, the transcript levels of *GI* and *FKF1* significantly increased in the *cop10-4* mutant compared with WT (Figure 2A,B). The timing of the peaks in *CO* and *FT* transcript levels did not change in *cop10-4* mutants. However, the *cop10-4* mutants showed higher expression of *FT*, although *CO* expression at ZT6 did not differ in *cop10-4* mutants compared with WT (Figure 2C,D).

Our results show that in *cop10-4* mutants, the expression levels of *GI* and *FT*, as well as *FKF1*, were significantly up-regulated during daytime in SD conditions (Figure 2A,D). By contrast, *CO* expression did not differ in *cop10-4* mutants compared with WT (Figure 2C), indicating that COP10 delays flowering by down-regulating the *GI-FT* regulatory module in non-inductive SD conditions. Interestingly, it appears that the expression patterns of *GI*, *FKF1*, and *CO* in *cop10-4* mutants differ considerably from those in *det1-1* mutants [22]. Under SD conditions, the expression levels of *GI* and *FKF1* did not significantly change in *det1-1* mutants compared with WT. By contrast, the *CO* transcript levels significantly increased in *det1-1* mutants, compared with WT [22]. As COP10 and DET1 form a complex together with DDB1 (CDD complex) [33], we expected that the expression levels of key flowering genes, such as *GI*, *FKF1*, and *CO*, were similarly regulated in *cop10-4* and *det1-1* mutants. However, our results indicate that the COP10- and DET1-dependent mechanisms regulating flowering in SD differ somewhat, and that COP10 probably regulates flowering in SD without forming the CDD complex. 

In this experiment, we focused on the transcriptional regulation of key flowering genes in *cop10-4* mutants. However, COP10 is a subunit of the E3 CUL4-CDD E3 ligase [26]. Thus, it is also important to examine whether the protein levels of key flowering factors, such as GI, FKF1, CO, and FT, were altered in *cop10-4* mutants, which will be investigated in future work.

### 2.3. COP10 Interacts with GI

**Figure 3:**
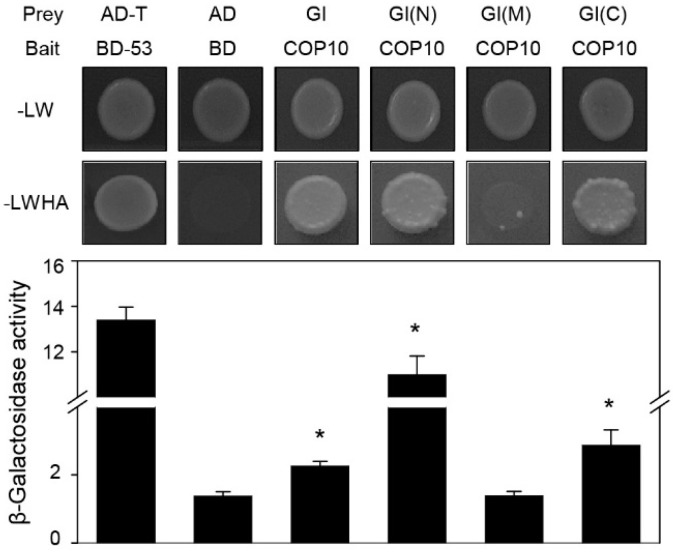
COP10 directly interacts with GI in a yeast two-hybrid assay. The bait was full-length COP10. For prey, GI was divided into three pieces: N-terminal (N; aa 1–507), middle (M; aa 401–907), and C-terminal (C; aa 801–1173). pGBKT7-53 (BD-53) and pGADT7-T were used as a positive control. Empty pGBKT7 (BD) and pGADT7 (AD) vectors were the negative control. SD medium (-LWHA; lacking tryptophan, leucine, histidine, and adenine) was used to select for the interaction between baits and preys. β-Galactosidase assays were performed according to the manufacturer’s protocol. Means and standard deviations were obtained from three individual colonies. Asterisks indicate statistically significant differences compared to negative control as determined by Student’s *t*-test (* *p* < 0.05). These experiments were repeated twice with the same results.

DET1 physically interacts with GI, leading to decreased GI binding to the promoter of *FT* [22]. Although we found that mutation of *COP10* causes up-regulation of *GI* expression in SD conditions (Figure 2), it is also possible that COP10 physically associates with GI to down-regulate its function in flowering, similar to DET1. To test this possibility, we first examined the physical interaction between COP10 and GI by yeast two-hybrid assays. We used GI for the prey and COP10 for the bait. Yeast cells transformed with *COP10* and *GI* grew on SD-4 selection media lacking adenine, leucine, histidine, and tryptophan. Especially, yeast cells, in which COP10 was co-transformed with constructs expressing the full-length, N-terminal (aa 1–507), and C-terminal (aa 801–1173) regions of GI, but not the middle region (aa 401–907), grew on the interaction media. We additionally performed chlorophenol red-β-d-galactopyranoside (CPRG) assays to qualitatively measure these interactions, which revealed that COP10 interacts most strongly with the N-terminal (aa 1–507) region of GI (Figure 3).

**Figure 4:**
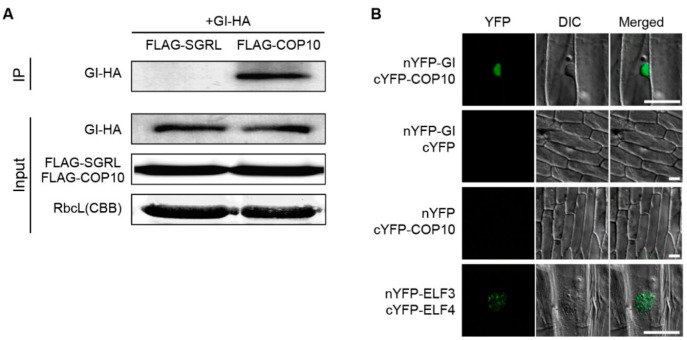
COP10 interacts with GI in plants. (**A**) Co-immunoprecipitation of COP10 and GI using *35S:FLAG-COP10*, *35S:HA-GI*, and *35S:SGRL-FLAG*. Total protein was extracted from two-week-old seedlings. FLAG-beads were used for pull-down. Anti-HA antibody was used for GI-HA protein band. *35S:SGRL-FLAG* plants served as a negative control. The upper panel is co-immunoprecipitated GI-HA protein using an anti-HA antibody after an anti-FLAG-bead pull-down assay. As a loading control, RbcL protein levels were visualized by staining the immunoblot with Coomassie Brilliant Blue; (**B**) BiFC analysis of the interaction between COP10 and GI in the nucleus of an onion epidermal cell. nYFP-ELF3 and cYFP-ELF4 plasmids served as a positive control [21]. For the negative controls, empty nYFP-GI/cYFP and nYFP/cYFP-COP10 were used. Each pair of recombinant plasmids encoding nYFP and cYFP fusions was mixed 1:1 (*w*/*w*) and co-bombarded into onion epidermal cell layers. The transformed onion epidermal layers were incubated at 22 °C for 16–24 h under dark condition. YFP fluorescence was indicated by green color. Scale bar = 50 μm. These experiments were repeated three times with the same results.

To test the *in vivo* interaction of COP10 and GI, we subsequently performed co-immunoprecipitation (co-IP) assays using transgenic plants overexpressing *HA-GI* or *FLAG-COP10*. FLAG-tagged STAY-GREEN LIKE (SGRL-FLAG), a chloroplast-localized protein [34], was used for a negative control. *35S:FLAG-COP10*, *35S:HA-GI*, and *35S:SGRL-FLAG* (a negative control) transgenic plants were grown for 2 weeks in SD, and then sampled at ZT8. We found that HA-GI co-immunoprecipitated with FLAG-COP10, but not with SGRL-FLAG (Figure 4A). To further confirm this *in vivo* interaction, we performed bimolecular fluorescence complementation (BiFC) assays using onion epidermal cells. COP10 and GI localize in the cytosol and nucleus, respectively [25,34]; thus we also investigated where they interact. Using a transient expression assay in onion epidermal cells, we detected strong reconstituted YFP fluorescence in the nucleus when *nYFP-GI* and *cYFP-COP10* plasmids were co-transformed, but we detected no YFP fluorescence in the onion cells when we co-transformed *nYFP-GI/cYFP* or *nYFP/cYFP-COP10* (Figure 4B). Taken together, these results indicate that COP10 physically interacts with GI *in vitro* and *in vivo*, and this interaction occurs in the nucleus.

To date, the significance of this COP10-GI interaction remains unclear, but we can consider a few possibilities. We previously found that DET1 physically interacts with GI in the nucleus to decrease GI binding to the promoter of *FT* for repression of the induction of flowering [22]. DET1 and COP10, together with DDB1, also form a CDD complex in the nucleus to repress photomorphogenesis [33]. Thus, COP10 could act with DET1 to modulate GI binding to the *FT* promoter. COP10 also acts as an enhancer of a ubiquitin-conjugating E2 enzyme and interacts with COP1, a known a RING E3 ubiquitin ligase, in the ubiquitination pathway [25,33]. Furthermore, we previously suggested that COP1 may destabilize GI protein by interacting with EARLY FLOWERING 3 (ELF3) [23]. Thus, COP10 (in the CDD complex or another form) could also function with COP1 to control GI in the photoperiod pathway. Further biochemical analyses of the interaction between GI and COP10 will be necessary to reveal the significance of the GI-COP10 interaction.

### 2.4. COP10 Regulates FLOWERING LOCUS C (FLC) Expression through Interaction with MULTICOPY SUPPRESSOR OF IRA1 4 (MSI4) in the Autonomous Pathway

**Figure 5:**
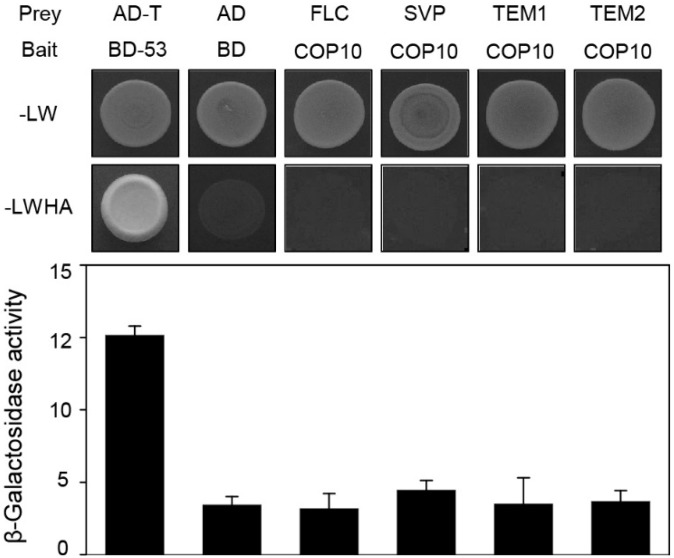
COP10 does not interact with *FT* repressors in yeast. The bait was full-length COP10. For prey, FLC, SVP, TEM1, and TEM2 were used. pGBKT7-53 (BD-53) and pGADT7-T were used as a positive control. Empty pGBKT7 (BD) and pGADT7 (AD) vectors were the negative control. SD medium (-LWHA; lacking tryptophan, leucine, histidine, and adenine) was used to select for the interaction between bait and prey proteins. β-Galactosidase activity assays were performed according to the manufacturer’s protocol. Means and standard deviations were obtained from three individual colonies. These experiments were repeated twice with the same results.

**Figure 6:**
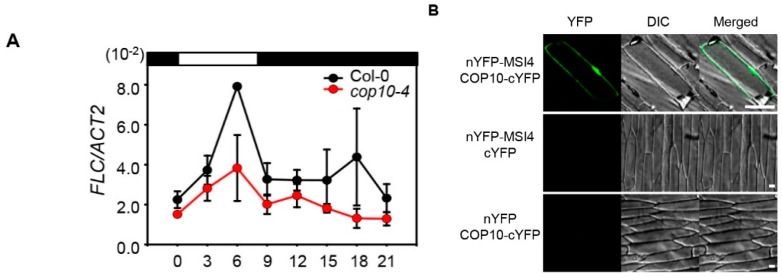
COP10 regulates *FLOWERING LOCUS C* (*FLC*) expression through interaction with MULTICOPY SUPPRESSOR OF IRA1 4 (MSI4)/FVE (MSI4/FVE). (**A**) The expression of *FLC* analyzed in Col-0 and *cop10-4* mutants by real-time PCR using three-week-old plants. Plants were grown at 22 °C under SD (8-h light:16-h dark) conditions, and plant tissues were harvested every 3 h. *ACT2* expression was used for normalization. Means and standard deviations were obtained from three biological replicates. ZT, zeitgeber time (h after dawn); (**B**) COP10 interacts with MSI4 in plants. BiFC analysis of the interaction between MSI4 and COP10 in onion epidermal cells. nYFP-ELF3 and cYFP-ELF4 plasmids served as a positive control [21]. For the negative controls, empty nYFP-GI/cYFP and nYFP/cYFP-COP10 were used. Each pair of recombinant plasmids encoding nYFP and cYFP fusions was mixed 1:1 (*w*/*w*) and co-bombarded into onion epidermal cell layers. The transformed onion epidermal layers were incubated at 22 °C for 16–24 h under light conditions. YFP fluorescence was indicated by green color. Each bar indicates 50 μm. DIC, differential interference contrast. These experiments were repeated three times with the same results.

The expression of *FT* is intricately regulated; GI and CO activate *FT* transcription and FLC, SVP, TEM, and TEM2 repress *FT* transcription [13,14,15,35]. GI directly interacts with SVP, TEM1, and TEM2, probably to deactivate or destabilize these *FT* repressors [35]. Therefore, COP10 could act with these *FT* repressors to down-regulate *FT* expression. Thus, we examined the possible interaction of COP10 and repressors of *FT* transcription in yeast two-hybrid assays. These revealed that COP10 does not interact with FLC, SVP, TEM1, or TEM2 (Figure 5), indicating that COP10 likely does not directly affect the activity of these *FT* repressors. We next used RT-qPCR to examine whether mutation of *COP10* alters the expression levels of these *FT* repressors in WT and *cop10-4* mutants grown in SD conditions. We found that *FLC* mRNA levels significantly decreased in *cop10-4* mutants compared with WT (Figure 6A). By contrast, levels of *SVP*, *TEM1*, and *TEM2* mRNAs did not significantly differ in *cop1-4* mutants and WT (Figure S1).

*FLC* expression is regulated in the autonomous pathway, which consists of several factors involved in RNA processing and epigenetic regulation [36]. MSI4/FVE acts as a key regulator of the autonomous pathway to reduce *FLC* expression [37]. Furthermore, both DET1 and DDB1, members of CDD complex, interact with MSI4/FVE to reduce its activity and consequently up-regulate *FLC* expression [20,22]. Thus, COP10 could also interact with MSI4, as well as DET1 and DDB1, possibly in a CDD complex. To examine this possibility, we used BiFC assays to examine the physical interaction between COP10 and MSI4/FVE. Using transient expression in onion epidermal cells, we detected strong reconstituted YFP fluorescence in both cytosol and nucleus when *nYFP-MSI4* and *COP10-cYFP* plasmids were co-transformed (Figure 6B).

DET1 participates in chromatin remodeling by interacting with unmodified histone H2B [38]. Furthermore, the CDD complex acts in chromatin remodeling with CUL4 through the interaction with non-acetylated H2B, to repress photomorphogenesis under light conditions [39,40]. A recent study showed that MSI4/FVE interacts with HDA6 and directly binds to the *FLC* locus to repress *FLC* transcription via chromatin remodeling [19,20]. In this scenario, the CDD complex and MSI4/FVE act in the autonomous pathway by histone modification of the *FLC* promoter with HDA6. Our findings provide evidence that COP10 also has an important role in regulating *FLC* expression through interaction with MSI4/FVE in the autonomous pathway, possibly as a part of the CDD complex. In this study, we also found by BiFC analysis that COP10 physically interacts with MSI4/FVE in both the cytosol and nucleus (Figure 6A). COP10 originally localizes in the cytosol and MSI4/FVE l localizes to the cytosol and nucleus [25,37]. Thus, MSI4/FVE likely acts in the translocation of COP10 from the cytosol to nucleus. Many circadian-clock and flowering components localize in the nucleus and the cytosol, and this subcellular compartmentalization has important roles in the regulation of photoperiodic flowering. For example, the *Arabidopsis* flowering regulator EARLY-FLOWERING4 (ELF4) acts as a regulator of the nucleus/cytosol distribution of GI through a physical interaction [41]. Analogous to ELF4, it is possible that the subcellular compartmentalization of COP10 is also regulated by the interaction with MSI4/FVE or other components in flowering pathway, which will be investigated in more detail in future work.

## 3. Experimental Section

### 3.1. Plant Materials and Growth Conditions

*Arabidopsis thaliana* WT and mutant lines used in this study are in the Columbia (Col-0) genetic background. The *gi-1* seeds (CS3123) were obtained from the Arabidopsis Biological Resource Center (Columbus, OH, USA). The *cop10-4* mutants were obtained from Xing Wang Deng. *cop10-4* has been analyzed as one of the *cop10* mutant alleles, which has a mutation in the second exon of *COP10*, resulting in a weak allele [25]. To create *cop10-4 gi-1* double mutants, F_1_ heterozygotes were obtained by crossing the *cop10-4* mutant as the female plant with *gi-1* mutants as pollen donors. Plants were grown on soil at a constant 22 °C under white fluorescent light (90–100 μmol·m^−2^·s^−1^) in LD (16-h light:8-h dark) and SD (10-h light:14-h dark) conditions.

### 3.2. Analysis of Flowering Time

Flowering time was measured by counting the total number of rosette leaves (RLs) at bolting. Data were obtained from three experimental replications (20 plants per replication).

### 3.3. RNA Preparation, Reverse Transcription, and Quantitative Real-Time PCR (qPCR) Analysis

Green tissues of 3-week-old WT and *cop10-4* plants grown on agar plates (containing 4.3 g/L Murashige Skoog, Duchefa Biochemie, Haarlem, The Nederland) under LD or SD conditions were collected every 3 h. Total RNA was extracted with the MG Total RNA Extraction Kit (Macrogen, Seoul, Korea) according to the manufacturer’s instructions. For each sample, 2 μL of total mRNA was reverse-transcribed using oligo (dT)_15_ primers and M-MLV reverse transcriptase (Promega, Madison, WI, USA). The levels of the transcripts were measured by the relative quantification method, using GoTaq qPCR Master Mix (Promega) and a Light Cycler 480 (Roche Applied Science, Penzberg, Upper Bavaria, Germany). The qPCR conditions were: 95 °C for 2 min, and then 45 cycles of 95 °C for 10 s and 60 °C for 1 min. Each qPCR amplification was repeated at least three times with biologically independent samples for statistical analysis. *ACTIN2* (*ACT2*) was used for an internal control. The amount of each mRNA was determined using specific primers (Table S2).

### 3.4. Yeast Two-Hybrid Assays

Yeast two-hybrid assays were performed according to the Yeast Protocols Handbook (Clontech, Mountain View, CA, USA). The full-length cDNA of *COP10* was amplified from WT total RNA using RT-PCR, and cloned into the pGBKT7 (bait) vector. The full-length cDNAs of *FLC*, *SVP*, *TEM1*, and *TEM2*, and three partial fragments of *GI* (encoding the N-terminal, aa 1–507; middle aa 401–907; and C-terminal, aa 801–1173) were cloned into the pGADT7 (prey) vector (MATCHMAKER GAL4 two-hybrid system 3, Clontech) as described previously [21,35]. For the interaction study, plasmids expressing fusion proteins were transformed into the yeast (*Saccharomyces cerevisiae*) strain AH109 [42] by the LiAc-mediated method [43] and grown on media lacking adenine, leucine, histidine, and tryptophan. Chlorophenol red-β-d-galactopyranoside (CPRG; Roche Biochemicals, Penzberg, Upper Bavaria, Germany) was used to measure the β-galactosidase activity according to the manufacturer’s protocol.

### 3.5. In Vivo Pull-Down Assays

*Arabidopsis* (Col-0 ecotype) plants were used in this study. The transgenic plants containing the *35S:FLAG-COP10* or *35S:HA-GI* constructs were previously described [33,44]. In addition, *35S:SGRL-FLAG*, which we previously generated [45], was used as a negative control. For the COP10-GI interaction assays, *35S:FLAG-COP10*, *35S:HA-GI*, and *35S:SGRL-FLAG* plants were grown on 0.5× Murashige-Skoog (MS) phytoagar medium in SD (8-h light:16-h dark) for 10 days and then vacuum infiltrated for 10 min in MS liquid medium (Duchefa Biochemie, Duchefa Biochemie, Haarlem, The Nederland) supplemented with 50 mM MG132 (Sigma-Aldrich, St. Louis, MO, USA) for proteasome inhibitor treatment. After treatment, plants were incubated for 10 h under light conditions, and then homogenized; total proteins were extracted in total protein extract buffer (50 mM Tris-HCl (pH 7.5), 100 mM NaCl, 10 mM MgCl_2_, 1 mM EDTA (pH 8.0), 10% glycerol, 1 mM PMSF, 1 mM DTT). These experiments were performed with FLAG-M2 magnetic beads (Sigma-Aldrich) for FLAG-IP. After washing, the immunoprecipitated fractions were determined by immunoblot analysis. The HA-GI fusion proteins were immunodetected by an anti-HA antibody (Cell Signaling Technology, Danvers, MA, USA).

### 3.6. Bimolecular Fluorescence Complementation Assays in Onion Epidermal Cells

The full-length cDNA of *COP10* was cloned into the BiFC gateway vectors [46] to examine COP10 *in vivo* interactions. Cloned vectors containing *GI*, *ELF3*, *ELF4*, and *MSI4* were previously prepared [22,23]. For partial YFP-tagged *COP10* and *MSI4* constructs, the cDNA of each gene was obtained by RT-PCR from WT (Col-0) plants and fused into four BiFC plasmid sets, pSAT5-DEST-cEYFP(175-end)-C1(B) (pE3130), pSAT5(A)-DEST-cEYFP(175-end)-N1 (pE3132), pSAT4(A)-DEST-nEYFP(1-174)-N1(pE3134), and pSAT4-DEST-nEYFP(1-174)-C1 (pE3136) to generate constructs fused with the N- or C-terminal fragments of YFP. Partial YFP-tagged *ELF3* and *GI* constructs were previously described [23]. Purified gold particles were coated with each pair of recombinant plasmids encoding nEYFP and cEYFP fusions, which were mixed 1:1 (*w*/*w*), and co-bombarded into onion epidermal layers using a DNA Particle Delivery System (Biolistic PDS-1000/He, BioRad, Hercules, CA, USA). The transformed onion epidermal cells were incubated on MS solid media with 50 mM MG132 (Sigma-Aldrich) for 16–24 h at 22 °C. Onion cells used in Figure 4 (nYFP-GI/cYFP-COP10 and positive/negative control) and in Figure 6 (nYFP-MSI4/COP10-cYFP and positive/negative controls) were incubated under dark and light conditions, respectively. These experimental conditions were selected because *nYFP-GI/cYFP-COP10* was predominantly expressed in dark-incubated cells while *nYFP-MSI4/COP10-cYFP* was predominantly expressed in the light. Then, the fluorescence was detected using a confocal laser scanning microscope (Carl Zeiss LSM710, Germany). nYFP-ELF3/cYFP-ELF4 served as a positive control [22], and empty nYFP-GI/cYFP and empty nYFP/cYFP-COP10 were used for the negative control.

### 3.7. Accession Numbers

Sequence data from this article can be found in the *Arabidopsis* Genome Initiative or GenBank/EMBL databases under the following accession numbers: *CO* (At5g15840), *COP10* (At3g13550), *ELF3* (At2g25930), *ELF4* (At2g40080), *FKF1* (At1g68050), *FLC* (AT5G10140), *FT* (At1g65480), *GI* (At3g13550), *MSI4* (At2g1952), *SVP* (At2g22540), *TEM1* (At1g25560), and *TEM2* (At1g68840).

## 4. Conclusions

**Figure 7:**
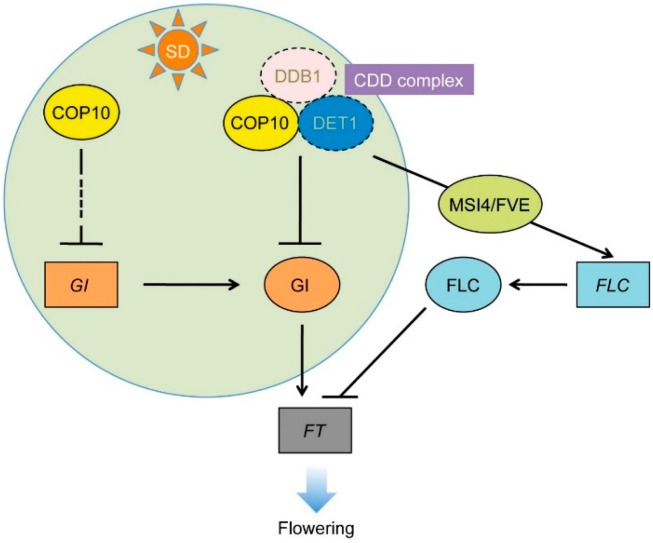
Working model of COP10 function in flowering repression*.* COP10 suppresses *FT* expression via multiple routes in the photoperiod and autonomous pathways. In the photoperiod flowering pathway, COP10 functions in floral repression by regulation of GI at the transcriptional and post-translational levels during daytime under SD. COP10, probably without forming the CDD complex, blocks formation of the FKF1-GI complex by repressing expression of *FKF1* and *GI* during the daytime. Second, COP10 physically interacts with GI, and might repress its binding to the *FT* promoter. In the autonomous pathway, COP10 modulates *FLC* chromatin through interaction with MSI4 to induce *FLC* expression. Genes and proteins are represented as rectangles and ovals, respectively.

Here we showed that COP10 negatively regulates the SD-dependent flowering pathways. Because COP10 and DET1 act together in the CDD complex [33], we expected COP10 and DET1 to act similarly in the regulation of SD-dependent repression of flowering. However, we found that in SD conditions, *GI* and *FKF1* expression levels were significantly upregulated in *cop10-4* mutants, but we observed no change in *CO* expression level (Figure 2). These expression patterns are quite different from those in the *det1-1* mutant [22], suggesting that COP10 and DET1 may regulate the main cascades of photoperiodic flowering differently, and probably not as part of the CDD complex (Figure 7). On the other hand, we found that mutation of *COP10* decreases the expression of *FLC* (Figure 6A), probably due to the lack of interaction of COP10 with MSI4/FVE, a key regulator of the autonomous pathway (Figure 6B). Furthermore, COP10 directly interacts with GI *in vitro* and *in vivo* (Figure 3 and Figure 4). Such a mode of action of COP10 is considerably similar to DET1 [22]. Thus, it is possible that COP10 and DET1 act together, in the CDD complex, for posttranslational regulation of the function of GI and the MSI4-FLC cascade in the autonomous pathway (Figure 7). However, further experiments, such as expression analysis of flowering time genes in *det1-1 cop10-4* double mutants and time course co-IP of COP10, DET1, and GI, will be needed to understand the role of the CDD complex in repression of flowering pathways.

## Supplementary Materials

Supplementary materials can be found at http://www.mdpi.com/1422-0067/16/11/25969/s1.
