# Supplementary material for: CONSTITUTIVE PHOTOMORPHOGENIC 10 (COP10) Contributes to Floral Repression under Non-Inductive Short Days in Arabidopsis
Source: Int J Mol Sci. 2015 Nov 5;16(11):26493–505. doi: 10.3390/ijms161125969 (PMC4661828; doi:10.3390/ijms161125969)
Supplement: Supplementary file 1 [file ijms-16-25969-s001.pdf]

International Journal of
Molecular Sciences

MDPI

Supplementary Information

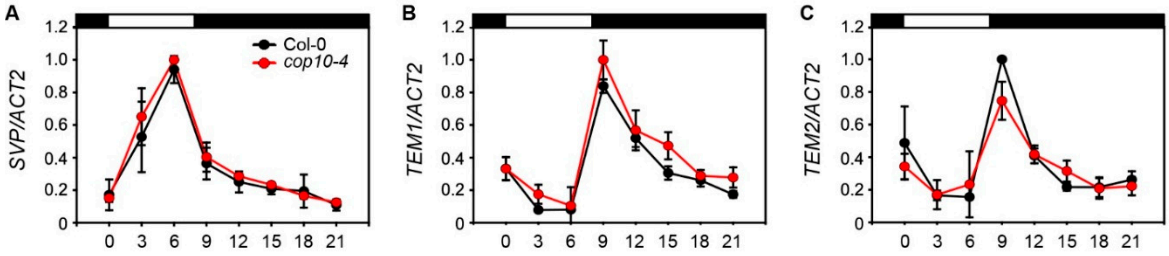

Figure S1. Effect of *cop10-4* on *SVP*, *TEM1*, and *TEM2* expression under SD. The expression of *SVP* (**A**); *TEM1* (**B**); and *TEM2* (**C**) was analyzed in Col-0 and *cop10-4* mutants by real-time PCR using 3-week-old plants. Plants were grown at 22 °C under SD (8-h light:16-h dark) conditions, and plant tissues were harvested every 3 h. *ACT2* expression was used for normalization. Means and standard deviations were obtained from three biological replicates.

Table S1. Effect of *cop10-4* mutation on flowering time in LD and SD.

Genotype	Rosette Leaves at Bolting	
	LD (16-h L:8-h D)	SD (10-h L:14-h D)
Wild type (Col-0)	10.8 ± 0.9	44.3 ± 4.9
cop10-4	10.7 ± 1.5	34.0 ± 2.8
gi-1	32.1 ± 3.7	51.2 ± 2.2
gi-1 cop10-4	32.1 ± 1.8	50.6 ± 1.9

LD, long day; SD, short day; L, light period; D, dark period.

Table S2. Primers used in this study.

Name	Sequence (5′→3′)	Application
CO_F	GCCTACTTGTGCATGAGCTG	Real-time PCR
CO_R	GTTTATGGCGGGAAGCAAC	Real-time PCR
FKF1_F	GTTGTACCGCCTCCAAGACT	Real-time PCR
FKF1_R	AGATGATGACCCTACCACACG	Real-time PCR
FLC_F	GCTACTTGAACTTGTGGATAGCAA	Real-time PCR
FLC_R	GGAGAGGGCAGTCTCAAGGT	Real-time PCR
FT_F	GGTGGAGAAGACCTCAGGAA	Real-time PCR
FT_R	GGTTGCTAGGACTTGGAACATC	Real-time PCR
GI_F	TGCATCTGGTGTAAGGCTACC	Real-time PCR
GI_R	CCTATAGCCCGCAAGAAGTG	Real-time PCR
ACT2_F	TGGGATGAACCAGAAGGATG	Real-time PCR
ACT2_R	AAGAATACCTCTCTTGGATTGTGC	Real-time PCR
COP10_F	GAATTCATGATGACACCTGGCGGAAG	Y2H, BiFC
COP10_R	GGATCCTCACTTGGCAAATCGCAATG	Y2H, BiFC
FLC_F	GAATTCATGGGAAGAAAAAAACTAG	Y2H
FLC_R	GGATCCCTAATTAAGTAGTG	Y2H
SVP_F	GAATTCATGGCGAGAGAAAAGATTCA	Y2H

Table S2. *Cont.*

Name	Sequence (5′→3′)	Application
SVP_R	GGATCCCTAACCACCATACGGTAA	Y2H
TEM1_F	GAATTCATGGAATACAGCTG	Y2H
TEM1_R	GGATCCTCACAAGATGTTGA	Y2H
TEM2-F	CATATGATGGATTCTAGTTGCATAGA	Y2H
TEM2-R	GAATTCTCACAAAGCATTGATTATC	Y2H
ELF4-F	CCAGGTATTGCTGATAGAATGAG	Y2H, BiFC
ELF4-R	CTGAGGGAAGCCAAGATAGAG	Y2H, BiFC
MSI4_F	ATGGAGAGCGACGAAGCAG	BiFC
MSI4 _R	TTAAGGCTTGGAGGCACAAGTCA	BiFC
